# Pluripotent Stem Cell-Derived Human Tissue: Platforms to Evaluate Drug Metabolism and Safety

**DOI:** 10.1208/s12248-017-0171-8

**Published:** 2017-12-21

**Authors:** Jose Meseguer-Ripolles, Salman R. Khetani, Javier G. Blanco, Mairi Iredale, David C. Hay

**Affiliations:** 1 MRC Centre for Regenerative Medicine, 5 Little France Drive, Edinburgh, EH16 4UU, UK; 2 University of Illinois at Chicago, Bioengineering (MC 063) 851 S Morgan St, 218 SEO, Chicago, Illinois 60607, USA; 3 School of Pharmacy and Pharmaceutical Sciences, University at Buffalo, The State University of New York, Buffalo, New York, USA

**Keywords:** drug development, heart, liver, pluripotent stem cell, toxicity

## Abstract

Despite the improvements in drug screening, high levels of drug attrition persist. Although high-throughput screening platforms permit the testing of compound libraries, poor compound efficacy or unexpected organ toxicity are major causes of attrition. Part of the reason for drug failure resides in the models employed, most of which are not representative of normal organ biology. This same problem affects all the major organs during drug development. Hepatotoxicity and cardiotoxicity are two interesting examples of organ disease and can present in the late stages of drug development, resulting in major cost and increased risk to the patient. Currently, cell-based systems used within industry rely on immortalized or primary cell lines from donated tissue. These models possess significant advantages and disadvantages, but in general display limited relevance to the organ of interest. Recently, stem cell technology has shown promise in drug development and has been proposed as an alternative to current industrial systems. These offerings will provide the field with exciting new models to study human organ biology at scale and in detail. We believe that the recent advances in production of stem cell-derived hepatocytes and cardiomyocytes combined with cutting-edge engineering technologies make them an attractive alternative to current screening models for drug discovery. This will lead to fast failing of poor drugs earlier in the process, delivering safer and more efficacious medicines for the patient.

## INTRODUCTION

Despite improvements in drug screening, there is still a high percentage of drug attrition during development. This presents in either in pre-clinical modeling, clinical trials or after drug approval, with greater expense incurred the further along the pipeline the compounds are removed. Therefore, fast failing is key to improving the success and the cost of human drug development. The percentage of drug failure at phase II and phase III is high and the main reasons for failure are the lack of efficacy, 48% in phase II and 55% in phase III, and safety, 25% in phase II and 14% in phase III (1). A recent study analyzed the main reasons for a drug withdrawn from the market because of adverse effects from 1950 to 2014. Hepatotoxicity (18%) represented the first reason for drug withdrawal followed by immune-related reactions (17%) and with cardiotoxicity third (14%). Hepatotoxicity and cardiotoxicity represent serious concerns in drug development. Side toxic effects are often detected at later stages of the development or even after the drug approval. Because of that, there is a need to improve current screening models to improve the early detection of hepatotoxic and cardiotoxic drugs (2). Although high-throughput screening platforms permit the testing of large compound libraries during drug development, the high attrition rates demonstrate the need for improved screening platforms and more reliable pre-clinical models. An essential component of this is to improve model fidelity (for a detailed review see (3)). Key to this is our ability to recapitulate organ physiology ‘in the dish’. Improvements in this space will likely lead to improved safety, efficacy and reduced development costs (3).

Current cell-based models used within industry rely heavily on immortalized cell lines, usually derived from human tumors. These models have advantages, such as cost-effective scale up and well to well consistency. Additionally, these cell lines are amenable to genetic engineering, permitting gain and loss of function analysis. While these models demonstrate advantages, they offer limited biological relevance when compared to the intact organ and primary cell types. Currently, primary cells and tissue slices are the gold standard for drug discovery, as they exhibit greater resemblance to the organ of interest. There are however drawbacks with these resources. The main disadvantages of using primary cell types or tissue slices are their labor intensive isolation from diseased organs, the scarcity of donor tissue, the rapid loss of cell phenotype, and significant batch to batch variation (4).

Stem cell technology has shown promise in drug screening (5,6) and has been proposed as a suitable alternative to overcome the above-mentioned limitations with primary cell types. Current advances in embryonic stem cell (ESC) and induced pluripotent stem cell (iPSC) differentiation protocols better mimic primary cells than the immortalized lines (7). This, in combination with enabling techniques such as 3D culture, microfabrication, fluid flow, and cell encapsulation, offers the prospect of more accurate models to study organ biology. Through model refinement and cost-effective scale up, it is now possible to prototype systems for drug development scientists from defined genetic backgrounds to study and better understand the biology behind idiosyncratic drug-induced liver injury (8–10). The power of these systems in combination with label free technologies and multiparametric data analysis offer exciting prospects for the future (3,6,11,12). In this concise review, we will highlight some past and present efforts in the field.

## HUMAN DRUG METABOLISM

The liver plays a central role in drug disposition; it is responsible for drug uptake, metabolism and excretion. Several factors are involved in the off-target effects of drugs with differential metabolism playing a key role. Cytochrome P450 enzymes play an important roles during drug metabolism, with five family members (CYP1A2, CYP2C9, CYP2C19, CYP2D6, and CYP3A4) responsible for the metabolism of approximately 90% of marketed drugs. Genetic polymorphisms in the CYP450 family members affects drug metabolism, efficacy and safety (13,14). Polymorphisms in phase II enzymes, such as UDP-glucuronosyltransferases, N-acetyltransferases, and glutathione S-transferases, and ABC transporters are also known to influence metabolism and drug exposure (for a review please see (14)). Following drug metabolism, the metabolites are an important concern. Those can be active and provide patient benefit; however, they also expose the organ to adverse events, including endoplasmic reticulum stress (19,20). This can lead to alterations in cell signaling pathways that can alter the cell fate upon toxic insult such as NF-κB and Nrf-2 (15,16). Mitochondrial stress is also evident, altering cellular ATP and reactive oxygen species levels triggering cell death pathways (17,18). Drug metabolism and cell stress are therefore key concerns during the drug development process to reduce possible side effects of new drugs.

Detection of potential cardiotoxic drugs is also important due to the percentage of heart disease that exists in the population (21). Drug metabolites can cause cardiotoxicity via changes in action potentials and altered ion channel activity. The most common drug-induced cardiotoxicity is the Torsade de Pointes (TdP), a type of ventricular arrhythmia caused by ion cahnnel blocking. Additionally, the Ether-à-go-go-Related Gene (hERG) channel is commonly blocked by drug interaction which can result in the development of long QT syndrome (22). Notably, multiple drugs can alter QT interval prolongation increasing the risk of cardiac failure, including anticancer drug metabolites (23,24).

The factors discussed above are important considerations for drug development. One major consideration is the diversity of the population, which is not captured by many in vitro models. This requires the development of more sophisticated systems for human drug development. We believe that stem cell-based technologies have the power to capture variability observed in the population. Stem cell-derived hepatocytes and cardiomyocytes have been used successfully to study drug metabolism on defined genetic backgrounds, providing critical proof of concept that pluripotent stem cell-derived cell types are enabling for human drug development (for reviews see 25 and 26).

## HEPATOCYTE SCREENING MODELS

The gold standard model for the study of drug metabolism during drug development is the primary hepatocyte. The main disadvantages of primary hepatocytes are their rapid loss of phenotype post isolation and isolation costs (4 23). Therefore, researchers have searched for more accessible and cheaper alternatives. Cancer-derived cell lines, such as HepG2, HuH-7, Hep3B, or Fa2N-4, and HepaRG have been used to characterize some determinants of dug metabolism (24,27,28). Pluripotent cell-derived models have been proposed as an alternative cell source for screening (6,29,30). In many cases, pluripotent cell-derived models exhibit drug sensitivities patterns similar to primary cells (5,7,31–33). Moreover, the use of pluripotent stem cells allows the user to derive somatic cells from defined background, thereby offering insight into idiosyncratic DILI (34,35). To date, most of the work has focused on monolayer hepatocyte systems derived from induced pluripotent stem cells, through defined and reproducible differentiation protocols. Despite these advances, monolayer cultures of hepatocytes face significant limitations and do not emulate the complexity of the liver in terms of tissue organization, blood flow, and different cell-cell interactions. To overcome these limitations, organoid or spheroid models have been developed showing promising results (36,37). Although they require more complex differentiation protocols, organoids/ spheroids better recapitulate human tissue structure and display more mature and functional phenotype such as improved cytochrome P450 3A4 activity, greater expression of Phase II and III enzymes, combined with reduced fetal gene expression and longer lifespan (38). While promising the current challenges that face the B3D field^ is cost-effective manufacture, experimental reproducibility and automated scale-up for application.

### Hepatocyte Differentiation from Pluripotent Stem Cells

Several groups have established differentiation protocols that allow the efficient differentiation of human pluripotent stem cells into hepatocyte-like cells (HLCs). Hepatocyte differentiation attempts to recreate aspects of human liver development using growth factors and small molecules (5,12,39–50)(summarized in Table I).

**Fig. 1:**
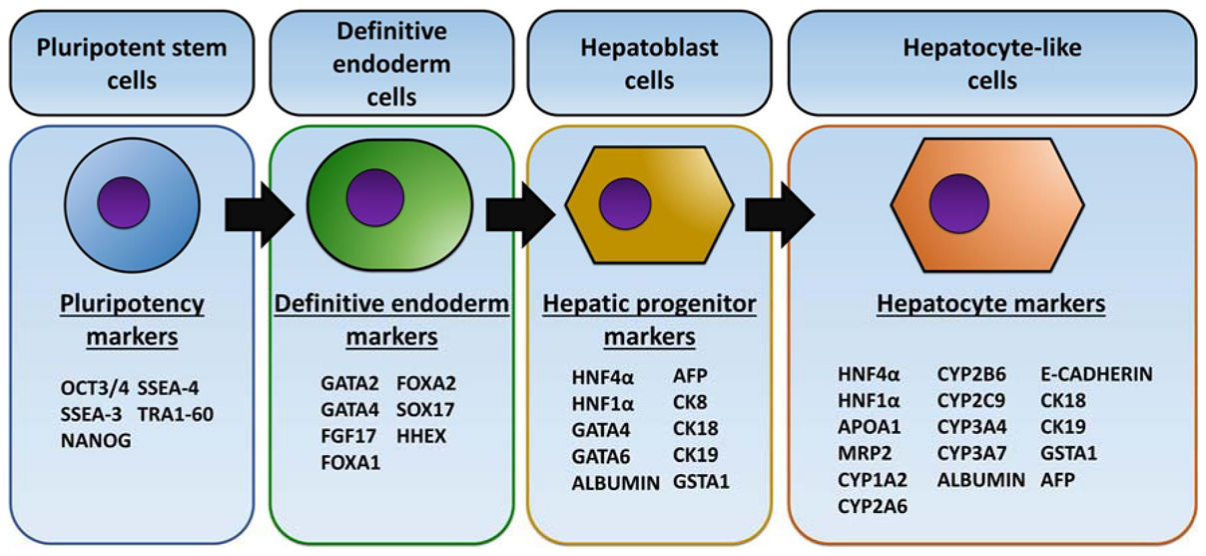
Stagewise differentiation of pluripotent stem cells to hepatocyte-like cells. Pluripotent stem cells are differentiated to definitive endoderm, then primed to the hepatoblast stage. Following this, the progenitors are matured to hepatocyte-like cells. A panel of markers can be employed to assess successful differentiation at each stage of the process. Those include; OCT3/4 - octamer-binding transcription factor 4, SSEA-4—stage-specific embryonic antigen 4, SSEA-3—stage-specific embryonic antigen 3, GATA2 - GATA binding protein 2, GATA4—GATA binding protein 4, GATA6—GATA binding protein 6, FOXA2—forkhead box protein A2, FOXA1—forkhead box protein A1, FGF17—fibroblast growth factor 17, hHex—hematopoietically-expressed homeobox, HNF4α—hepatocyte nuclear factor 4 alpha, HNF1α—hepatocyte nuclear factor 1 alpha, AFP—alpha-fetoprotein, CK8—cytokeratin 8, CK18—cytokeratin 18, CK19—cytokeratin 19, GSTA1—glutathione S-transferase A1, APOA1—apolipoprotein A1, MRP2—multidrug resistance-associated protein 2, CYP1A2—cytochrome P450 1A2, CYP2A6—cytochrome P450 2A6, CYP2B6—cytochrome P450 2B6, CYP2C9—cytochrome P450 2C9, CYP3A4—cytochrome P450 3A4, CYP3A7—cytochrome P450 3A7

Hepatocyte monolayer differentiation systems usually consist of stagewise approach where the stem cell populations are driven to definitive endoderm using growth factors such as, activin A and Wnt3a (41,51) (Fig. 1). This is followed by hepatic progenitor cell specification (42,43,49) and hepatocyte maturation (39,44–48,50,52) (Fig. 1). These protocols produce HLCs that express hepatocyte markers such as HNF4a, albumin, and cytochrome P450 proteins (Fig. 1) (6,8,29,30). Advantages of the 2D systems include the automated and cost effective scale up, and limited batch variation, making them ideal prototypes for drug screening. However, 2D systems do face some limitations, such as the mixture of fetal and adult hepatocyte traits, limited tissue structure and as a consequence cannot recapitulate all situations that occur in vivo (53).

### HLCs as a Tool for Disease Modeling and Drug Screening

Recent advances in the last decade have demonstrated the potential of HLCs as a tool to model human diseases and drug exposure and some examples follow. Rashid et al. (46) and Cayo et al. (54) produced HLCs from patient-derived human iPSC cell lines which accurately modeled human metabolic liver disease. Similarly, Graffman et al. developed a system to study non-alcoholic fatty liver disease from human pluripotent stem cells, inducing fat storage in HLCs and detecting dysregulated expression of metabolism-associated genes (55). More recently, a study performed by Kim et al. (56) provided proof of concept that HLCs can predict drug-induced hepatotoxicity in which the immune system played a role. HLCs secreted pro-inflammatory cytokines and chemokines, such us TNF-α, interleukin1β, interferon α, and chemokine (C-C motif) ligand 5, which activated immune cell lines. Additionally, Lucendo-Villarin et al. (57) showed how HLCs can be used to study fetal hepatotoxicity when exposed to tobacco derivatives. Relative to other types of hepatocyte sources, HLCs were found to be more sensitive than the cancer cell line HepG2 (58) and exhibit a comparable response to primary hepatocytes when challenged with toxins (33,58), suggesting that HLCs are suitable for drug screening. Drug repurposing is another potential use of stem cell-based technologies. While continued research into the cell niche is required to further improve the HLC phenotype, the studies described above evidence the power of stem cell-derived HLCs to model human disease and improve drug discovery.

In addition to the above, collections of iPSC-derived cell lines from multiple donors would facilitate studies aimed to determine whether Bdonor specific^ factors modify the pharmacological profile of drug candidates. For example, panels of human iPSC-derived HLCs with distinct CYP450 genotypes would be useful to uncover potentially significant sources of interindividual variability in drug metabolism. However, widespread implementation of this attractive paradigm is limited by difficulties inherent to the production and characterization of relatively large numbers of iPSC-derived cell lines (59). Furthermore, it remains to be determined whether the impact of Bdonor specific^ factors such as functional genetic polymorphisms and epigenetic signatures is closely reproduced within the milieu of iPSC-derived cell types. In this regard, a recent study by Takayama et al. showed that HLCs exhibit interindividual differences in drug metabolism that are similar to the ones found in the originating primary human hepatocytes (PHHs) (31). The authors generated 12 individual HLCs cell lines and performed extensive comparisons with the originating PHHs by using an array of phenotypic assays. Notable findings includedCYP1A2, CYP2C9, and CYP3A4 activities between HLCs vs PHHs exhibited good correlation (r^2^ > 0.7); HLCs and PHHs displayed similar CYP2C9-mediated metabolism for the hepatotoxic substrate benzbromarone; and the impact of CYP2D6 polymorphic variants on CYP2D6 activity was comparable between HLCs and the originating PHHs. The authors noted that future studies should examine whether HLCs differentiated from other cell can also recapitulate interindividual differences in drug metabolism.

### Engineering Approaches to Improve HLC Differentiation and Maturation

Randomly distributed 2D cultures/co-cultures containing primary human hepatocytes (PHHs) and HLCs are straight-forward to create but do not allow control over homotypic/ heterotypic cell-cell interactions and cell-ECM signaling that are known to affect liver functions in vivo. In contrast, several engineering tools, such as cellular microarrays, protein micropatterning, microfluidics, biomaterial scaffolds, and bioprinting, now allow precise control over the cellular microenvironment to enhance hepatocellular function. Long-term (4+ weeks) stabilization of function typically requires co-cultivation with liver- or non-liver-derived non-parenchymal cell types (52). For instance, Berger et al. developed a micropatterned co-culture platform (iMPCC) in which human iPSC-derived HLCs were organized into collagen-coated domains of empirically optimized dimensions and subsequently surrounded by 3 T3-J2 murine embryonic fibroblasts (60), a cell type that expresses molecules present in the liver (61 62). In contrast to phenotypically declining HLC monolayers, iMPCCs displayed high and stable liver functions (i.e., CYP450 enzyme activities) and a significant reduction in fetal markers (i.e., alpha-fetoprotein) for 4+ weeks. Ware et al. subsequently treated iMPCCs in 96-well plates with 47 drugs for 6 days, and evaluated function/ viability endpoints (albumin, urea, and ATP) over time (32). Results showed 65% sensitivity (24 of 37 hepatotoxic drugs) and 100% specificity (9 of 9 non-liver-toxic drugs) in iMPCCs, which were remarkably similar to the sensitivity/ specificity (70%/100%) in MPCCs containing PHHs treated with the same drugs for 5–9 days (63). Such studies suggest iMPCC utility for an initial hepatotoxicity screen in early drug development.

In addition to the MPCC platform, PHHs (64), stem cell-derived HLCs (65,66), and adult human liver bipotential cells (67) can be differentiated and stabilized in 3D spheroids/ organoids, which leads to the establishment of homotypic cell-cell interactions and the presence of ECM proteins within and around the cells. Hepatic spheroids can spontaneously form on non-treated culture plates or those coated with various polymers (64,68). Such spheroids/organoids have been shown to display high viability/functions and in vivo-like responses to drugs (64); however, it is difficult to control the spheroid size and smaller spheroids can merge to form larger spheroids with necrotic cores due to poor diffusion of oxygen/nutrient. To mitigate such a challenge, specialized plates and scaffolds have been developed to direct the assembly of uniformly sized spheroids that remain separated for interrogation following drug/stimuli treatment. For instance, Takayama et al. utilized a nanopillar plate to create HLC spheroids, which were more sensitive to drug toxicity than HepG2 spheroids (65); however, HLCs spheroids displayed lower sensitivity than conventional PHH monolayers, suggesting that further maturation of the HLCs is likely required. Similarly, Tasnim et al. encapsulated human pluripotent stem cell-derived hepatocyte-like cells in galactosylated cellulosic sponges, which promoted the formation and retention of spheroids (69); such spheroids were more sensitive to the toxicity of hepatotoxic drugs as compared to conventional monolayers, and responses in stem cell spheroids were like those observed in PHHs. The above-mentioned approaches to form spheroids typically result in a randomly distributed/ heterogeneous architecture. In contrast, Ma et al. utilized bioprinting to create liver lobule-like hexagonal organoids containing HLCs, endothelial cells, and adipose-derived stem cells embedded in a hydrogel (70). Liver gene expression and functions in co-cultured organoids were detected for ~ 32 days at higher levels than in monocultures. Ultimately, standardization of protocols to form spheroids via different approaches, as well as cost reduction, will be required for routine deployment in drug development.

The engineering approaches described above improve HLC function, but do not always elucidate the microenvironmental signals (and their combinations) underlying the observed responses. On the other hand, cellular microarrays, in which viable cells are seeded onto printed spots of materials/biomolecules, represent a powerful approach for precisely defining the optimal microenvironment of cells (71–75). Cellular microarrays based on spotted biomaterial libraries have been applied to several investigations aimed at exploring stem cell functions on changing polymer backbone chemistries and end-group functionalization (76–80). For instance, Kaylan et al. utilized an ECM microarray approach to demonstrate that ECM composition exhibits a significant influence on the adhesion and degree of differentiation of mouse liver progenitor cells when they are induced to differentiate (81). We anticipate that the use of cellular microarrays for defining precise molecular conditions for HLC differentiation will continue to grow in this field.

## STEM CELL-DERIVED CARDIOMYOCYTES

### Differentiation of Cardiomyocytes from Pluripotent Stem Cells

In 2006 and 2007, Takahashi and Yamanaka demonstrated that the introduction of specific factors into differentiated fibroblasts, of fetal and adult origin, induce cellular reprogramming into PSCs (82,83). The generation of iPSCs from differentiated fibroblasts is achieved through viral expression of the transcription factors such as Oct4, Sox2, Klf4, and cMyc. Further differentiation of iPSC into cardiomyocytes can be performed using several methods, including the traditional embryoid body formation, activation/inhibition of signaling pathways with specific proteins, and modulation of the Wnt signaling pathway via small molecule regulators. Most of the current protocols for the generation of cardiomyocytes from iPSC result in mixtures of ventricular, atrial and nodal-like cardiomyocytes (84).

Cardiomyocytes derived from iPSCs maintain an immature phenotype in culture. For example, iPSC-derived cardiomyocytes are small, have irregular shapes, and exhibit lower membrane capacitance in comparison to mature cardiomyocytes. Structurally, iPSC-derived cardiomyocytes are mono-rather than bi- or multi-nucleated cells that lack T-tubules, have disarrayed sarcomeres with immature patterns of myofibrillar isoforms, and have relatively low mitochondrial content. iPSC cardiomyocytes use glucose instead of fatty acids as a metabolic substrate. iPSC-derived cardiomyocytes and mature cardiomyocytes also exhibit differences in relevant electrophysiological parameters (e.g., upstroke velocity, and resting membrane potential), contractile force, Ca^2+^ handling properties, and response to beta adrenergic stimulation (84). Several approaches are being tested to induce phenotypic maturation of iPSC-cardiomyocytes. These strategies are based on long-term culture, the use of topographical and biochemical cues, and co-culture with other cell types (85). Exploitation of the full potential of the iPSC-cardiomyocyte model for research and clinical applications still requires the development of strategies for the induction of mature cellular phenotypes. These strategies need to be reproducible, amenable to large scale culturing and screening applications, and suitable to produce Bclinical grade^ cells.

### iPSC-Derived Cardiomyocytes in Pharmaceutical Development

iPSC-derived cardiomyocytes are an attractive model for pharmaceutical research applications. The use of iPSC-derived cardiomyocytes in the context of pharmaceutical applications can be divided in two broad categories: (1) screening of drug candidates to identify compounds with cardiotoxic potential, and (2) in vitro modeling of cardiac diseases for the identification and validation of pharmacological targets. In general, cultures of iPSC cardiomyocytes are suitable for medium to high-throughput assays and high content imaging. There are diverse functional screening platforms that provide a range of information derived from the analysis of either single cells or multiple cells per well (for a review please see (86)). Cellular parameters such as contractility, membrane depolarization, and motion are inferred from the analysis of various readouts derived from fluorescent and luminescent Ca^2 +^ indicators, small molecule voltage probes, patch clamp, multi-electrode arrays, impedance measurements, and bright field microscopy.

### Examples of the Use of iPSC-Derived Cardiomyocytes for Drug Toxicity Studies

The iPSC-derived cardiomyocyte model has become a popular platform for evaluating the impact of drugs on various cellular parameters relevant to cardiac physiology. Some examples include early work by Tanaka et al. investigating the effects of ion channel inhibitors and beta-adrenergic agonists on electrophysiological properties (e.g., field potential waveform) of human iPSC-cardiomyocytes (87). Work by Yokoo et al. demonstrated that drugs that affect cardiac beating frequency and contractility in the clinic (e.g., adrenaline, isoproterenol, procainamide, and verapamil) also modify beating parameters on iPSC-cardiomyocytes (88). Also, early work by Braam et al. described the development of Bchip-based^ approaches amenable to medium to high-throughput modalities to facilitate the evaluation of drugs by measuring changes in action potential in clusters of electrically connected iPSC-cardiomyocytes. The authors evaluated a range of cardioactive and non-cardioactive compounds; in general, the iPSC-cardiomyocyte model was a good predictor of clinical effects (89). In a landmark study, Moretti et al. derived patient-specific iPSC-cardiomyocytes from two patients (and two non-affected controls) with long QT-syndrome; patient-derived iPSC-cardiomyocytes exhibited electrophysiological alterations typical of the syndrome and had increased susceptibility to cathecolamine-induced tachyarrhythmia (90). The generation and evaluation of Blibraries^ of iPSC-cardiomyocytes from patients with different genetic cardiac disorders allows evaluating whether differences in susceptibility to cardiotoxic drugs are associated to the individual’s genetic background (91). This paradigm has been extended towards the characterization of molecular determinants for drug-induced cardiotoxicity in other clinical settings. For example, an interesting study by Burridge et al. showed that iPSC-cardiomyocytes derived from patients with breast cancer who developed anthracycline-related cardiotoxicity were more susceptible to doxorubicin toxicity than iPSC-cardiomyocytes from similarly treated patients who did not develop cardiotoxicity (92). Recent advancements in genome editing technologies will allow the generation of engineered iPSC-cardiomyocyte lines to examine the role of genetic variants during the development of specific types of drug cardiotoxicity.

## MOVING BEYOND CURRENT IN VITRO LIMITATIONS

Despite the advances, further refinement is required to better model the physiology of the organ of interest. Doing so will increase specificity and sensitivity of the screening models, thereby reducing the potential for off target drug events and failure. The ability to move beyond the current limitations requires interdisciplinary collaboration. By combining the best stem cell models with chemistry, physics, and engineering, new automated screening assays with improved function and physiology can be developed. Current areas of promise are discussed in the final section.

### Biomaterials and Scaffolds for In Vitro Model Maturation

Biomaterials and recombinant extracellular matrices have been shown to improve cell phenotype and cell maturity (93,94) in both hepatocytes (8) and cardiomyocytes (95) when cultured within 2D or 3D platforms. Organoid encapsulation by hydrogels such as alginate can be used for controlling the maturation, size and microenvironment of developing organoids (94,96). Moreover, hydrogels can be supplemented with specific ECM proteins or growth factors to mimic a specific tissue or disease environment for improved modeling. Current biomaterials have proven cost-effective and highly reproducible towards significantly reducing batch variation. Scaffolds can be obtained from natural materials such as laminins, alginate, or hyaluronic acid (8,95,97) or synthetic materials such as polyvinyl alcohol (PVA), polylactide-co-glycolide (PLG), poly [2-(methacryloyloxy) ethyl dimethyl-(3-sulfopropyl) ammonium hydroxide] (PMEDSAH) or poly (caprolactone) (PCL), (93,98).

### Automation and High Content Analysis as an Efficient Scale Up Production of ES-Derived Models

For screening proposes, stem cell differentiation procedures must be fully reproducible, display low levels of variation between wells and plates and be amenable to high content analysis and multiplexing. Automation of the protocols and a high-throughput assay development is pivotal for this; automated liquid handling systems allows the generation of large quantities of cells with reduced variation making them suitable for testing large compound libraries. Combining this with multiparametric profiling assays such as automated microscopy (99,100) or high-throughput genomics (101) allows the user to create a multi-parametric profile in response to the test compounds. Recent studies have probed the practical use of stem cell technology for high content screening for toxic drugs by a combination of high content microscopy and single endpoint assays (6,102). A clear example of the significance of these technologies is captured by the work of Bray at (103,104). They tested over 30,000 small molecules in a cell line using the Bcell paint^ assay, a multiplexed assay that allows in-depth morphological and organelle profiling at an acceptable cost. in vitro organ-on-chip for as a multiorgan toxicity model.

While hepatotoxicity and cardiotoxicity are critical issues to address during pharmaceutical drug development, drugs can also cause toxicity to other organ systems resulting in adverse outcomes. Thus, culture platforms that can adequately mimic organ-organ interaction upon drug exposure are also required. By combining microfluidics, micropatterning, and 3D cultures, it is now possible to model organ microenvironment and organ-organ interaction (105,106). Such Bhuman-on-a-chip^ systems modulate the physiological fluid shear stress and can maintain a constant delivery of oxygen to the system, while also enabling paracrine communication between multiple tissue types via the flowing culture medium. Recently, Maschmeyer et al. (107) created an organ-on-a-chip platform that allowed paracrine interaction of 4 tissue types, namely intestine, liver, skin and kidney. This platform was used to study ADME and repeated dose toxicity testing, providing proof of concept of such an approach. We anticipate that adaptation of organ-on-a-chip technology to stem-cell-derived differentiated cells will offer a powerful tool to study mechanism of drug target and off target effects before progressing to in vivo testing.

## CONCLUSION

We believe that with the recent advances in stem cell differentiation, scale up, and performance, it is possible to create more accurate human tissue models for drug development. Advances in stem cell biology over the last decade has provided the field with more accurate human cell-based models that recapitulate key aspects of human drug metabolism, with better precision than cancer lines. These systems also provide comparable activity to primary cells. While this is encouraging, further improvements are necessary to improve predictive power. Tissue engineering has already played an important role in this space, with organ-on-chip devices now available via several commercial sources. Future efforts in the field should focus on developing high-throughput multi-organ systems, capable of real-time monitoring and multiplexing to reduce costs and improve the quality of data output.

## Figures and Tables

**Table I:** A summary of the differentiation methodologies developed for hepatocyte like cell production from pluripotent stem cells

Substrate	Definitive endoderm induction	Days	Hepatic specification	Days	Hepatic maturation	Days	% Albumin + HNFa expression	CYP450 activity	References
MEFs	AA	3	FGF4, BMP2	5	OSM, HGF	5	70 (ALB)	CYP 2B1/2	Cai et al. 2007 (39)
					BSA, FGF4, HGF, OSM, Dex	2			
MEFs/Collagen I	AA, FBS, KOSR	5	FGF4, HGF, KOSR	3	+		67.4	NT	Agarwal et al. 2008 (40)
					FGF4, HGF, OSM, Dex	9			
Matrigel	AA, Wnt3A	3	1% DMSO, 20% KOSR	5	HGF, OSM, HC	9	90	CYP 1A2	Hay et al. 2008 (41)
								CYP 3A4	
MEFs	AA, FGF2	3	1% DMSO, HGF	8	Dex	3	55.5	CYP 1A2	Basma et al. 2009 (42)
								CYP 3A	
					HGF	5			
MEFs	AA	5	FGF4, BMP2	5	+		80	NT	Si-Tayeb et al. 2010 (43)
					OSM	5			
	AA, Wnt3A	3						CYP 1A2	
Matrigel	+		1% DMSO, 20% KOSR	5	HGF, OSM, HC	5	70–90	CYP 3A4	Sullivan et al. 2010 (44)
	AA	2							
Matrigel	AA, BMP4, FGF2	3	FGF10	3	FGF4, HGF, EGF	8	NQ	CYP 3A	Touboul et al. 2010 (45)
	AA, FGF2, BMP4, Ly294002	3							
	+								
Fibronectin	AA, FGF2, CHIR99021	1	AA	5	HGF, OSM	17	83	CYP 3A4	Rashid et al. 2010 (46)
	+								
	AA, FGF2	1							
MEFs	AA, Wnt3a, HGF	3	1% DMSO, 20% KOSR	5	HGF, OSM	7	NQ	CYP 3A4	Chen et al. 2012 (47)
Matrigel	AA, Wnt3a	3	1% DMSO, 20% KOSR	5	HGF, OSM, HC	9	90	CYP 1A2	Szkolnicka et al. 2014
								CYP 3A4	Rashidi et al. 2016 (5,48)
			FGF4, BMP2	4					
Matrigel	AA	3	+		OSM, Dex	8	NQ	CYP 2B6	Song et al. 2009 (49)
			HGF, KGF	6					
Laminin	AA, Wnt3a	3	1% DMSO, 20% KOSR	5	HGF, OSM, HC	9	90	CYP 1A2	Cameron et al. 2015
								CYP 3A4	Wang et al. 2017 (12,50)

MEFs mouse embryonic fibroblasts, AA activin A, Dex dexamethasone, OSM oncostatin M, FGF2 fibroblast growth factor 2, FGF4 fibroblast growth factor 4, FGF10 fibroblast growth factor 10, BSA bovine serum albumin, EGF epidermal growth factor, BMP2 bone morphogenic protein 2, BMP4 bone morphogenic protein 4, KGF keratinocyte growth factor, HGF hepatocyte growth factor, DMSO dimethyl sulfoxide, KOSR knockout serum replacement, FBS fetal bovine serum, HC hydrocortisone, CHIR99021–6-[[2-[[4-(2,4-dichlorophenyl)-5-(5-methyl-1 h-imidazol-2-yl)-2-pyrimidinyl]amino]ethyl]amino]-3-pyridinecarbonitrile, LY294002–2-(Morpholin-4-yl)-8-phenyl-4H-chromen-4-one, NQ expressed but not quantified, NT not tested
